# Midlife BMI and A Body Shape Index in relation to mortality and coronary heart disease events in a nationwide cohort

**DOI:** 10.3389/fnut.2026.1906316

**Published:** 2026-09-01

**Authors:** Itamar Shafran, Nir Y. Krakauer, Jesse C. Krakauer, Gali Cohen, Yariv Gerber

**Affiliations:** 1 Department of Epidemiology and Preventive Medicine, School of Public Health, Gray Faculty of Medical and Health Sciences, Tel Aviv University, Tel Aviv, Israel; 2 Department of Civil Engineering, City College of New York, New York, NY, United States; 3 Department of Endocrinology, Corewell Health William Beaumont University Hospital, Royal Oak, MI, United States

**Keywords:** A Body Shape Index, abdominal obesity, Body Mass Index, central obesity, cohort study, coronary heart disease, epidemiology, mortality

## Abstract

**Background:**

Obesity is a known cardiovascular risk factor. A Body Shape Index (ABSI) is a waist circumference (WC)-based measure of abdominal obesity, independent of Body Mass Index (BMI). We examined the independent and joint associations of ABSI and BMI with long-term mortality and coronary heart disease (CHD) events among initially young to middle-aged adults.

**Methods:**

Participants (*n* = 2,225) were drawn from the First Israeli National Health and Nutrition Survey (1999–2001), constituting adults aged 25–64. Baseline anthropometrics, including weight, height, and WC, were measured, enabling calculation of BMI and ABSI. Follow-up lasted through 2021 for mortality and 2022 for hospitalizations. Cox regressions assessed hazard ratios (HRs) for mortality and CHD hospitalization per 1-SD increase in the allometric indices, adjusted for socio-demographics, cardiovascular risk factors, and BMI or ABSI, as appropriate.

**Results:**

The baseline mean [SD] age was 43 [11] years, and 50% were women. Negligible correlation was observed between BMI and ABSI (*r* = 0.02). During follow-up, 247 deaths occurred, of whom 46 of CVD, and 267 were hospitalized for incident (first-ever) CHD. The multivariable-adjusted HRs for mortality were 1.06 (95% CI: 0.92–1.21) for BMI and 1.40 (95% CI: 1.21–1.63) for ABSI. ABSI exhibited a positive association across BMI categories; HR = 1.31 (95% CI: 0.98–1.77) in 18.5–24.9, 1.47 (95% CI: 1.16–1.86) in 25–29.9, and 1.35 (95% CI: 1.05–1.74) in BMI ≥ 30. The multivariable-adjusted HRs for CHD were 1.16 (95% CI: 1.01–1.34) for BMI and 1.43 (95% CI: 1.23–1.65) for ABSI, stronger in women.

**Conclusion:**

ABSI demonstrated stronger associations with mortality and CHD events than BMI and provided complementary prognostic information by identifying high-risk individuals in addition to BMI.

## Introduction

Cardiovascular disease (CVD) is a leading cause of morbidity and mortality worldwide, accounting for 17.9 million deaths per year, with one-third occurring in people under 70 years old, according to the World Health Organization (WHO).^1^ In the United States, CVD is the leading cause of mortality, with associated costs exceeding $250 billion in 2019–2020, according to the Centers for Disease Control and Prevention (CDC).^2^ A major and increasingly prevalent risk factor for CVD is obesity, with nearly 60% of the adult population worldwide classified as overweight or obese in 2022 (1). Obesity is strongly associated with multiple CVD-related conditions, including coronary heart disease (CHD), stroke, and heart failure (HF) (2). Notably, midlife obesity has been linked to increased risk of CVD-related hospitalizations and mortality in older age (3). While Body Mass Index (BMI) is commonly used to assess obesity, it has limitations. BMI does not distinguish between specific obesity phenotypes, such as abdominal or central obesity, which have been independently associated with increased mortality risk (4). To overcome this limitation, waist circumference (WC) is often used as a complementary measure of abdominal obesity (5). However, the strong correlation of WC with height and weight may limit its incremental value beyond BMI for risk stratification.

To address these limitations, A Body Shape Index (ABSI) was introduced in 2012 as a measure of WC adjusted for weight and height (6). Studies have demonstrated minimal correlation between ABSI and BMI, providing a distinct predictive value. ABSI was shown to be a strong independent predictor of various health outcomes, including frailty and mortality, regardless of BMI (7, 8). Although ABSI has been linked to cardiovascular risk in several populations, evidence regarding long-term CHD events and mortality in nationally representative cohorts remains limited. Given the rising prevalence of obesity and high CVD mortality rates in the general population, identifying reliable predictors of hospitalization and mortality risk has become increasingly pressing. We hypothesized that ABSI would be strongly associated with all-cause and CVD mortality, as well as CHD-related hospitalization, independent of BMI.

## Materials and methods

### Study design

In this cohort study, we assessed the associations of ABSI and BMI with all-cause mortality, CVD mortality, and CHD-related hospitalizations. The study was based on participants from the First Israeli National Health and Nutrition Survey, conducted between 1999 and 2001 by the Israel Center for Disease Control (ICDC) and the Nutrition Department of the Israeli Ministry of Health (MOH). The survey consisted of 3,246 participants aged 25–64 years, representative of the Israeli population. Participants were randomly selected from the national registry, with a 64.1% response rate.^3^ After excluding participants who were not successfully linked to registry outcome data (*n* = 438), had a BMI < 18.5 kg/m^2^ (*n* = 38), or had missing anthropometric data required to calculate ABSI (*n* = 551), the final study population consisted of 2,225 participants. Participants with BMI < 18.5 kg/m^2^ were excluded to minimize potential confounding from underlying illness or reverse causation related to illness-associated weight loss, consistent with a common approach in epidemiologic studies (9). We have compared basic socio-demographic and clinical factors between participants who were excluded from the study and those that were eventually included, suggesting that excluded participants tended to be female participants with lower income and non-Jewish ethnicity, but no other substantial differences (Supplementary Table 1).

### Allometric measurements

Height, body weight, and WC were measured at the beginning of the study. BMI was calculated as weight (kg) divided by height (meter, m) squared. As previously described in detail (6), ABSI was calculated as the ratio of measured WC (m) to the expected WC as a function of weight (kg) and height (m), as follows:


$$\text{ABSI}=(\mathrm{WC}*{\text{Weight}}^{-\frac{2}{3}}*{\text{Height}}^{\frac{5}{6}})=\frac{\mathrm{WC}}{{\mathrm{BMI}}^{\frac{2}{3}}*{\text{Height}}^{\frac{1}{2}}}$$

### Outcome registries

Outcome data were retrieved from TIMNA, a big data platform created by the Israeli MOH. The platform contains anonymized data derived from national registries, such as mortality, hospital admissions, procedures, and more. TIMNA links participants using their national identification numbers, which were not visible to the researchers (10).

### Mortality

Mortality data were retrieved from the National Death Registry (last updated December 2021), including the cause of death in the International Classification of Diseases 10th edition (ICD-10). Using the ICD-10 codes, we additionally categorized participants into CVD-related and non-CVD-related mortality. ICD-10 codes between I00-I99 were classified as CVD-related mortality causes.

### Hospitalizations

Using the National Hospitalization Discharge Registry (last updated December 2022), we retrieved each participant’s dates of admission and discharge, as well as the hospitalization’s diagnoses (documented in ICD-9 codes, stored as a primary diagnosis and secondary diagnoses) related to the hospitalization. Hospitalizations recorded before the study were also captured as the data in the registry dated back to January 1999 and were used to exclude participants with previous CHD-related hospitalizations from the hospitalization analysis. Both primary and secondary diagnoses were used to determine the cause of hospitalization, as was also done on previous studies based on the same registry (11, 12), with CHD-related hospitalization defined by ICD-9 codes 410–414 (13).

### Covariates

Baseline survey data included sociodemographic factors and clinical factors. Self-reported CVD risk factors, including hypertension, hyperlipidemia, diabetes (insulin- and non-insulin-dependent), smoking status, and physical inactivity, were obtained. We categorized marital status as married and single, with the latter including divorced, widowed, and separated. We classified ethnicity into three groups: Jewish, Arabs (including Arab Muslims and Christians), and other (including Druze and non-Arab Christians). Monthly income was divided into 10 categories. We grouped them into three categories: high (≥15,000 ILS), medium (6,001–15,000 ILS), and low (≤6,000 ILS). Smoking status was self-reported and classified as current, former, or never smoking. Data on various types of physical activity and their duration were reported. According to the American College of Sports Medicine Guidelines, we classified the participants into 3 activity levels (14, 15).

### Statistical analysis

For BMI and ABSI, we calculated hazard ratios (HRs) per 1 standard deviation (SD). Regression models of time to (1) all-cause death, (2) CVD death, and (3) incident (first-ever) CHD-related hospitalization were constructed using Cox proportional hazards regression models, with age as the time scale, where participants who survived until the end of follow-up were right censored. Because the primary aim was etiologic—to estimate the association of BMI and ABSI with mortality, CVD mortality, and the incident CHD-related hospitalizations—we used cause-specific Cox proportional hazards models, which estimate the event-specific hazard while treating death as a competing event. Participants with a history of CHD hospitalization before study entry were excluded from the CHD analysis. In a sensitivity analysis, we further excluded participants who self-reported a history of CVD. The proportional hazards assumption was tested using the Schoenfeld residuals, and no violations were found in any of the models. We used a set of four consecutive models for each assessed outcome, adjusting for additional covariates in turn. The first model used age as the time scale and was adjusted only for sex. The second model was further adjusted for socioeconomic factors: ethnicity, years of education, monthly income, and marital status. The third model was further adjusted for classic CVD risk factors: hypertension, hyperlipidemia, diabetes, smoking status, and physical inactivity. Finally, the last model was adjusted for the other allometric index (ABSI or BMI, as appropriate).

An additional analysis was stratified by sex to assess potential effect modification, and an additional analysis was preformed to detect potential interaction between sex and CHD-related hospitalizations. We used a missing indicator for variables with missing values exceeding 1%, including hypertension (2.6%) and monthly income (14.4%). Multiple imputation was performed as a sensitivity analysis. We also plotted HRs for mortality and CHD-related hospitalizations against the *Z* score for each anthropometric measure using the smoothHR package in R, in which continuous covariates were modeled as penalized splines, sex was held constant, and the reference value was set to the mean ABSI or BMI, as appropriate, allowing us to depict potential non-linear associations. Analysis was performed using R version 4.4.1 (The R Foundation for Statistical Computing).

## Results

The study included 2,225 participants, of whom 50.0% were women, with a mean (SD) age of 43 (11) years. Baseline characteristics across standard BMI levels and sample-based ABSI tertiles are shown in Table 1. Mean age increased with the BMI levels, together with the presence of cardiovascular risk factors, such as hypertension, hyperlipidemia, and diabetes. When stratifying participants by ABSI tertiles, mean age and the prevalence of cardiovascular risk factors increased with higher ABSI levels. Women were underrepresented in higher ABSI groups.

**Table 1:** Baseline characteristics across BMI and ABSI categories.

Variable	BMI Categories			*p*	SMD	ABSI Tertiles			*p*	SMD
	18.5–24.9	25.0–29.9	30+			Lower	Middle	Upper		
*N* (2,225)	835	901	489			742	741	742		
Age, mean (SD), year	39.4 (10.5)	44.5 (10.7)	46.9 (10.5)	<0.001	0.470	40.7 (10.5)	41.9 (10.6)	46.7 (10.9)	<0.001	0.374
Female, *n* (%)	460 (55.1)	377 (41.8)	275 (56.2)	<0.001	0.194	620 (83.6)	337 (45.5)	155 (20.9)	<0.001	1.006
Ethnicity, *n* (%)										
Jewish	679 (81.3)	679 (75.4)	348 (71.2)	<0.001	0.163	589 (79.4)	564 (76.1)	553 (74.5)	0.233	0.083
Arab	142 (17.0)	207 (23.0)	132 (27.0)			141 (19.0)	166 (22.4)	174 (23.5)		
Single status, *n* (%)	189 (22.6)	134 (14.9)	67 (13.7)	<0.001	0.156	144 (19.4)	148 (20.0)	98 (13.2)	0.001	0.122
Income, *n* (%)										
High	10 (1.2)	12 (1.3)	9 (1.8)	0.083	0.126	6 (0.8)	13 (1.8)	12 (1.6)	<0.001	0.280
Medium	152 (18.2)	197 (21.9)	94 (19.2)			92 (12.4)	141 (19.0)	210 (28.3)		
Low	562 (67.3)	570 (63.3)	299 (61.1)			524 (70.6)	480 (64.8)	427 (57.5)		
Education, mean (SD), year	13.64 (3.46)	13.04 (3.85)	12.02 (4.62)	<0.001	0.266	13.50 (3.70)	12.90 (3.76)	12.73 (4.30)	<0.001	0.130
Hypertension, *n* (%)	50 (6.0)	105 (11.7)	117 (23.9%)	<0.001	0.355	63 (8.5)	92 (12.4)	117 (15.8)	<0.001	0.153
Hyperlipidemia, *n* (%)	94 (11.3)	173 (19.2)	120 (24.5%)	<0.001	0.234	95 (12.8)	125 (16.9)	167 (22.5)	<0.001	0.172
Diabetes mellitus, *n* (%)	20 (2.4)	61 (6.8)	61 (6.8)	<0.001	0.262	18 (2.4)	50 (6.7)	73 (9.8)	<0.001	0.212
Smoking status, *n* (%)										
Current smoker	267 (32.1)	292 (32.4)	124 (25.5)	0.003	0.153	190 (25.6)	240 (32.5)	253 (34.2)	<0.001	0.265
Past smoker	124 (14.9)	157 (17.4)	108 (22.2)			93 (12.6)	128 (17.3)	168 (22.7)		
Non-smoker	442 (53.1)	451 (50.1)	254 (52.3)			458 (61.8)	370 (50.1)	319 (43.1)		
Physical activity, *n* (%)										
Inactive	461 (55.2)	483 (53.6)	294 (60.1)	0.125	0.102	360 (48.5)	419 (56.5)	459 (61.9)	<0.001	0.189
Insufficiently active	149 (17.8)	160 (17.8)	82 (16.8)			150 (20.2)	125 (16.9)	116 (15.6)		
Sufficiently active	221 (26.5)	256 (28.4)	109 (22.3)			231 (31.1)	194 (26.2)	161 (21.7)		
BMI, mean (SD), kg/m^2^						26.9 (5.14)	26.8 (4.62)	27.1 (4.19)	0.449	0.042
ABSI, mean (SD)	0.0780 (0.00575)	0.0792 (0.00597)	0.0785 (0.00602)	<0.001	0.135					

Low ABSI: 0.0543–0.0760; Moderate ABSI: 0.0760–0.0813; High ABSI: 0.0813–0.105.

ABSI, A Body Shape Index; BMI, Body Mass Index; SD, standard deviation; SMD, standardized mean difference.

Reported SMDs represent the average of all pairwise comparisons within each BMI and ABSI category.

**Figure 1:**
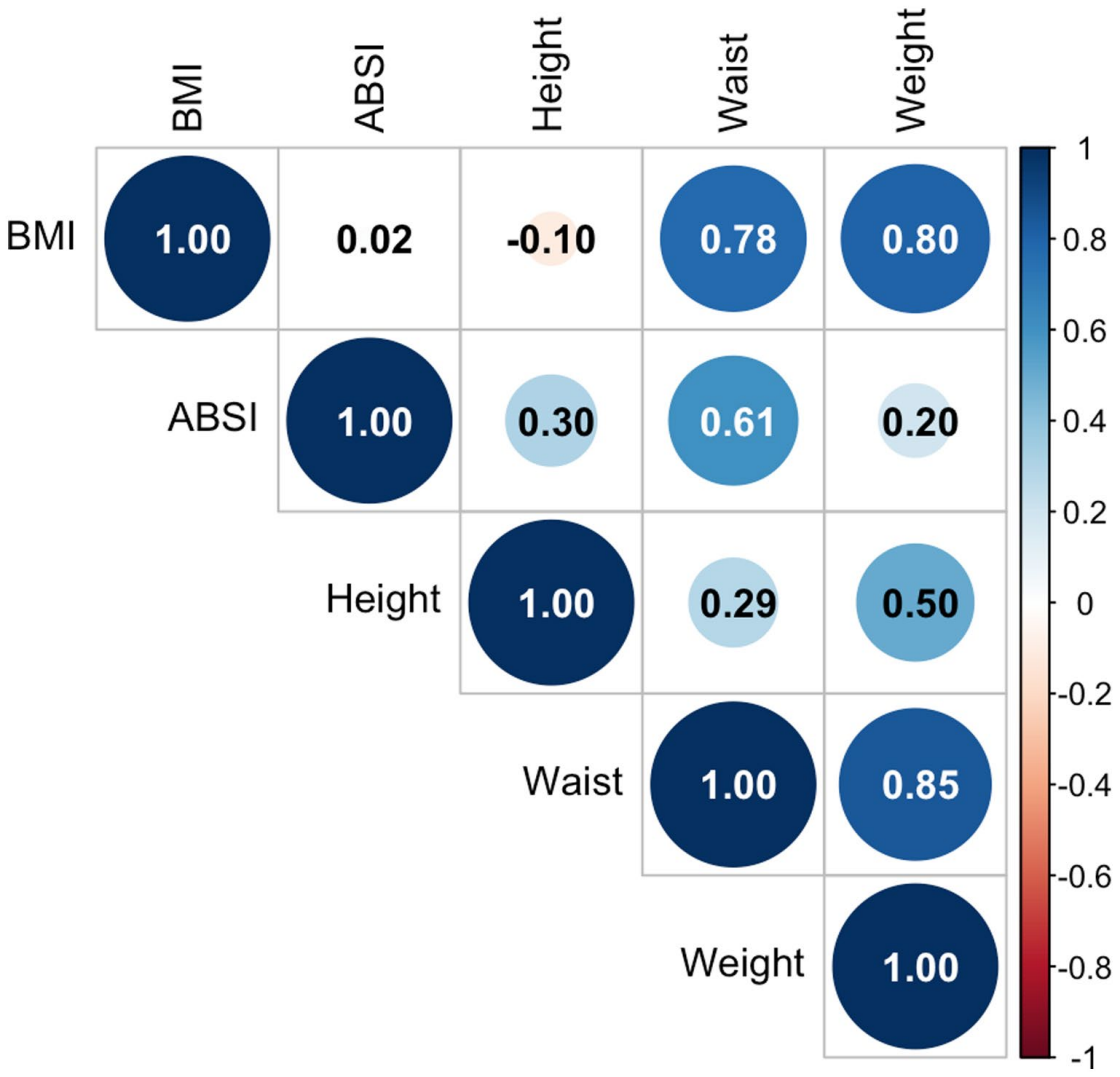
Correlation matrix between anthropometric measures. ABSI, A Body Shape Index; BMI, Body Mass Index.

We assessed the correlation coefficients between the anthropometric indices (Figure 1). The correlation between BMI and ABSI was nearly zero (*r* = 0.02). There was a strong correlation between BMI and WC (*r* = 0.78). ABSI showed a moderate correlation with height (*r* = 0.30).

### Associations of BMI and ABSI with all-cause mortality

After a median follow-up time of 21 years (IQR 21–22), 247 (11%) deaths were documented in the national registry, of whom 157 (63.56%) were among male participants. We calculated the HR for all-cause mortality per 1 SD increase in ABSI or BMI (Table 2). Increased HRs were shown in all adjusted models for ABSI, but not for BMI, which, after adjusting for socioeconomic and classic risk factors, was not significantly associated with increased mortality. Stratified by sex, the association between ABSI and mortality applied to both males and females. We further stratified the analysis by standard BMI categories. ABSI was consistently associated with overall mortality across all standard BMI categories, with HRs ranging from 1.32 to 1.50 (Table 3).

**Table 2:** Hazard ratios (95% confidence intervals) for all-cause mortality per 1 SD increase in ABSI and BMI.

Anthropometric		Adjustment			
		Sex	+ Socioeconomic factors	+ Classic risk factors	+ Anthropometrics
All-cause mortality					
BMI		1.15 (1.02–1.31)	1.06 (0.93–1.21)	1.04 (0.91–1.19)	1.05 (0.92–1.20)
ABSI		1.61 (1.40–1.86)	1.46 (1.27–1.68)	1.40 (1.21–1.62)	1.40 (1.21–1.63)
Stratification by sex					
Male (*n* = 1,113)	BMI	1.10 (0.90–1.33)	1.08 (0.88–1.31)	1.03 (0.83–1.27)	1.05 (0.85–1.29)
	ABSI	1.66 (1.35–2.03)	1.54 (1.26–1.87)	1.49 (1.22–1.82)	1.49 (1.22–1.82)
Female (*n* = 1,112)	BMI	1.19 (1.01–1.41)	1.03 (0.86–1.23)	1.05 (0.88–1.26)	1.07 (0.89–1.28)
	ABSI	1.57 (1.29–1.92)	1.39 (1.13–1.70)	1.25 (1.00–1.56)	1.26 (1.00–1.57)

HRs are reported per 1 SD increase (4.666 for BMI; 0.0059 for ABSI). Age was used as a timescale and was not adjusted for.

ABSI, A Body Shape Index; BMI, Body Mass Index; HR, hazard ratio; SD, standard deviation.

**Table 3:** Hazard ratios (95% confidence intervals) for the association between ABSI and all-cause mortality across BMI categories.

BMI category	Per 1 SD increase in ABSI
18.5–24.9 (*n* = 835)	1.32 (0.97–1.80)
25.0–29.9 (*n* = 901)	1.50 (1.18–1.90)
30.0 + (*n* = 489)	1.35 (1.04–1.74)

HRs are reported per 1 SD increase (0.0059 for ABSI) and adjusted to socioeconomic and classic risk factors.

ABSI, A Body Shape Index; BMI, Body Mass Index; SD, standard deviation.

**Figure 2:**
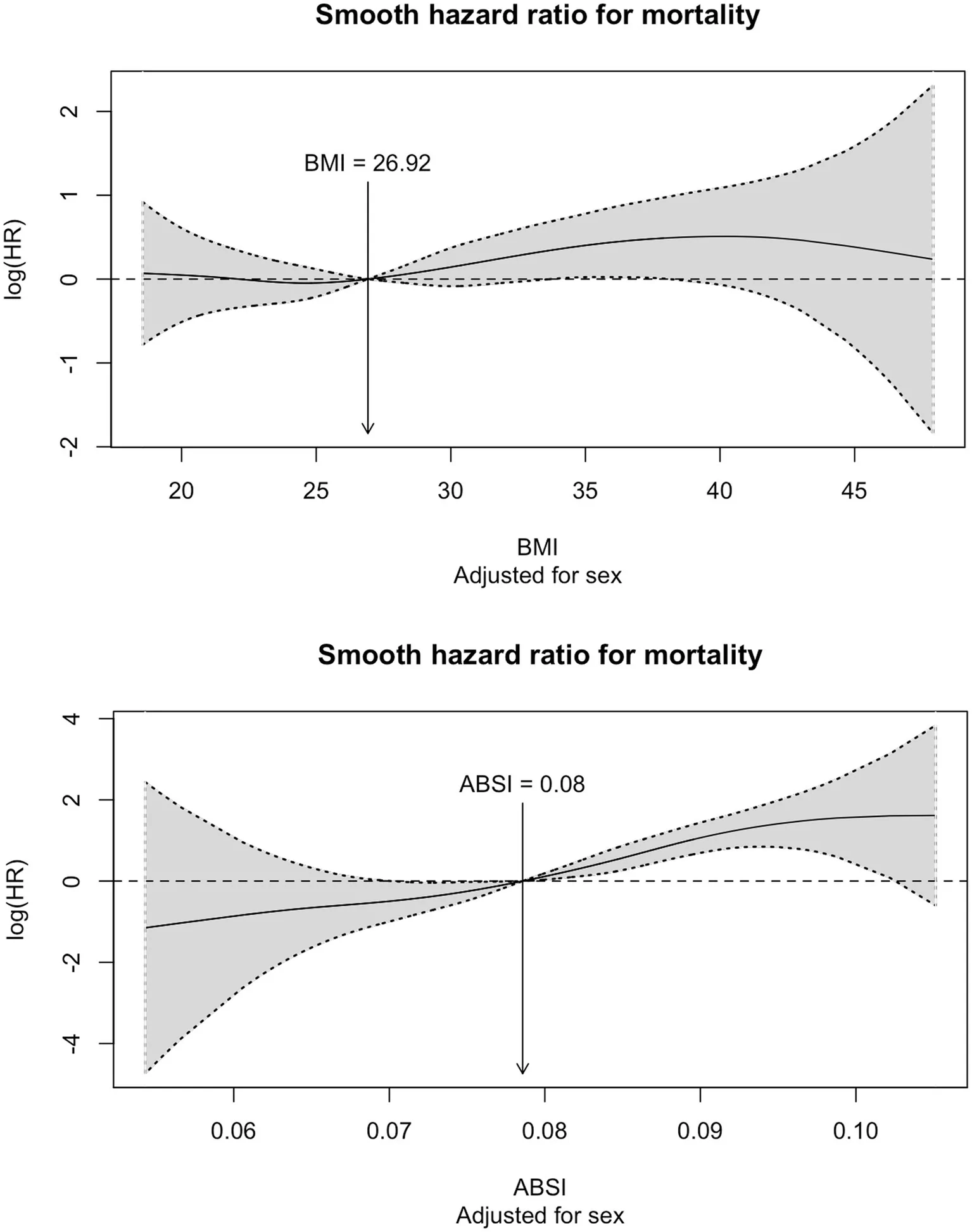
Adjusted smooth HR graphs for mortality. HR plotted against BMI or ABSI units and adjusted for sex; reference line set to mean value; ABSI, A Body Shape Index; BMI, Body Mass Index.

To assess potential non-linear associations between anthropometric measures and all-cause mortality, we plotted sex-adjusted log HRs across the distributions of BMI and ABSI, using the mean value of each measure as the reference. As demonstrated in Figure 2, BMI demonstrated a weak positive association for above-average BMI, whereas ABSI demonstrated a much stronger association, where the logarithm of mortality hazard increased approximately linearly with higher ABSI values.

### Associations of BMI and ABSI with CVD mortality

By the end of follow-up, of the 247 participants who died, 46 (19%) died of CVD. Among participants who died of CVD compared to non-CVD causes, there was no significant difference in mean baseline BMI levels (28.54 [SD 5.38] vs. 28.29 [SD 4.69], *p* = 0.77). In contrast, ABSI levels were significantly higher among CVD decedents compared with non-CVD decedents (0.0840 [SD 0.005] vs. 0.0821 [SD 0.006], *p* = 0.042). We estimated HRs for CVD and non-CVD mortality per 1 SD increase in BMI and ABSI. For BMI, associations with mortality were modest and attenuated after multivariable adjustment. In contrast, ABSI was consistently associated with increased risk of both CVD and non-CVD mortality across all models, with a stronger association observed for CVD mortality (Table 4).

**Table 4:** Hazard ratios (95% confidence intervals) for CVD and non-CVD mortality per 1 SD increase in ABSI and BMI.

Anthropometric		Adjustment			
		Sex	+ Socioeconomic factors	+ Classic risk factors	+ Anthropometrics
BMI	CVD	1.13 (0.85–1.52)	0.90 (0.65–1.23)	0.91 (0.67–1.25)	0.94 (0.68–1.30)
	Non-CVD	1.16 (1.01–1.33)	1.09 (0.93–1.11)	1.09 (0.93–1.26)	1.10 (0.95–1.29)
ABSI	CVD	2.14 (1.58–2.91)	1.72 (1.21–2.44)	1.79 (1.25–2.55)	1.78 (1.25–2.54)
	Non-CVD	1.50 (1.28–1.76)	1.36 (1.16–1.60)	1.33 (1.13–1.56)	1.34 (1.14–1.57)

HRs are reported per 1 SD increase (4.666 for BMI; 0.0059 for ABSI).

ABSI, A Body Shape Index; BMI, Body Mass Index; CVD, cardiovascular disease; HR, hazard ratio; SD, standard deviation.

### Associations of BMI and ABSI with CHD events

Of the 2,146 participants included in the CHD-related hospitalization analysis, 267 (12.4%) experienced their first CHD-related hospitalization during follow-up, of whom 201 (75.28%) were male participants and 66 (24.72%) were female. Compared with CHD-free participants, those hospitalized for CHD had significantly higher ABSI (0.082 [SD 0.006] vs. 0.078 [SD 0.006], *p* < 0.001) and BMI (28.1 [SD 4.69] vs. 26.7 [SD 4.62], *p* < 0.001). For both BMI and ABSI, elevated HRs were observed across all models. Stratified by sex, associations were generally stronger in women than in men and more pronounced for ABSI than for BMI (Table 5). We have further assessed the possible interaction between sex and BMI or ABSI, and the results suggest that after adjustments there was no interaction between sex and both ABSI and BMI (Table 6).

**Table 5:** Hazard ratios for CHD hospitalization per 1 SD increase in BMI and ABSI.

Anthropometric		Adjustment			
		Sex	+ Socioeconomic factors	+ Classic risk factors	+ Anthropometrics
CHD hospitalization					
BMI		1.28 (1.13–1.46)	1.21 (1.06–1.38)	1.16 (1.01–1.33)	1.17 (1.02–1.35)
ABSI		1.49 (1.29–1.72)	1.41 (1.23–1.63)	1.42 (1.22–1.64)	1.43 (1.23–1.65)
Stratification by sex					
Male (*n* = 1,058)	BMI	1.16 (0.98–1.38)	1.13 (0.94–1.35)	1.05 (0.87–1.27)	1.05 (0.87–1.27)
	ABSI	1.36 (1.14–1.63)	1.32 (1.11–1.57)	1.36 (1.14–1.64)	1.36 (1.14–1.63)
Female (*n* = 1,088)	BMI	1.40 (1.16–1.68)	1.29 (1.06–1.57)	1.29 (1.06–1.59)	1.39 (1.12–1.73)
	ABSI	1.74 (1.38–2.19)	1.59 (1.25–2.03)	1.57 (1.20–2.04)	1.68 (1.28–2.22)

HRs are reported per 1 SD increase (4.666 for BMI; 0.0059 for ABSI).

ABSI, A Body Shape Index; BMI, Body Mass Index; CHD, coronary heart disease.

**Table 6:** Sex and CHD-related hospitalizations interaction.

CHD hospitalization—sex interaction	Adjustment			
Anthropometric	Sex	+ Socioeconomic factors	+ Classic risk factors	+ Anthropometrics
BMI	*p* = 0.835	*p* = 0.170	*p* = 0.099	*p* = 0.110
ABSI	*p* = 0.039	*p* = 0.134	*p* = 0.419	*p* = 0.431

ABSI, A Body Shape Index; BMI, Body Mass Index; CHD, coronary heart disease.

**Figure 3:**
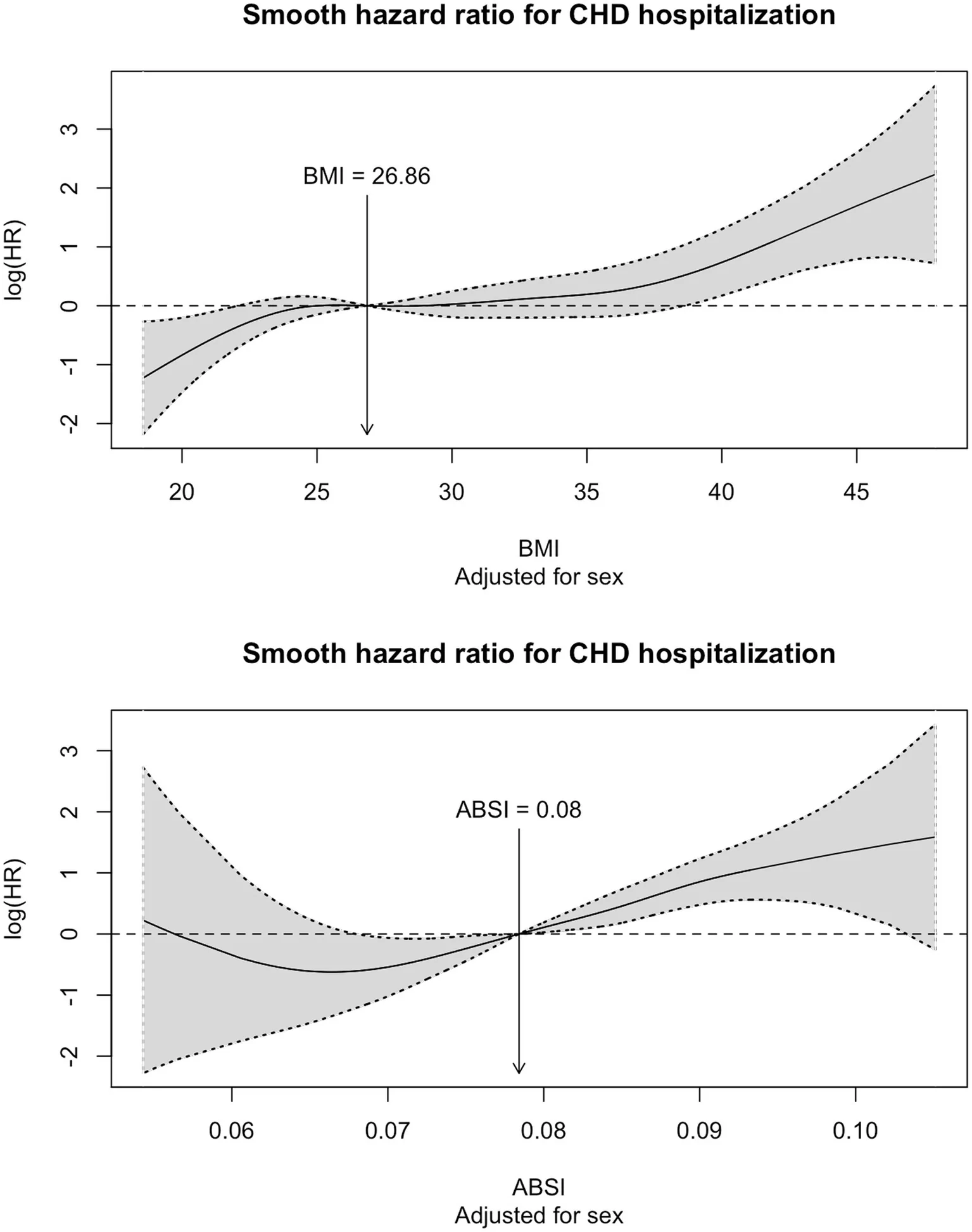
Adjusted smooth HR graphs for CHD hospitalization. HR plotted against BMI or ABSI units and adjusted for sex; reference line set to mean value; ABSI, A Body Shape Index; BMI, Body Mass Index.

Sex-adjusted HRs for CHD events were plotted against BMI and ABSI, using the sample mean as the reference value. BMI showed a generally linear positive association, with HRs increasing progressively at higher BMI values. For ABSI, a mostly linear increase in risk was observed above the mean, while a slight decline appeared at lower values. However, this decline was modest and based on a few participants, as reflected in the wider confidence intervals (Figure 3).

Several sensitivity analyzes supported the robustness of our findings. A multiple imputation analysis was preformed due to a monthly income missingness of 14.4%, assessing the HRs for all-cause mortality and CHD-related hospitalizations, adjusted for socioeconomic and classical risk factors, and was compared to the missing indicator method (Table 7). The results demonstrated similar HRs in both methods. Due to the possibility of missing previous hospitalization data in the registry, we preformed the analysis excluding self-reported CHD at study entry (additional 52 participants). The results were at large consistent with the primary analysis, where ABSI was a stronger predictor compared to BMI (Supplementary Table 2).

**Table 7:** Sensitivity analysis with missing indicator versus multiple imputation.

Anthropometric	Missing indicator HR	Multiple imputation HR
All-cause mortality		
BMI	1.04 (0.91–1.19)	1.03 (0.88–1.20)
ABSI	1.40 (1.21–1.62)	1.40 (1.18–1.65)
CHD-related hospitalizations		
BMI	1.16 (1.01–1.33)	1.17 (1.00–1.36)
ABSI	1.42 (1.22–1.64)	1.46 (1.24–1.72)

HRs are reported per 1 SD increase (4.666 for BMI; 0.0059 for ABSI) and adjusted to socioeconomic and clinical risk factors.

ABSI, A Body Shape Index; BMI, Body Mass Index; CVD, cardiovascular disease; HR, hazard ratio; SD, standard deviation.

## Discussion

In this large cohort of Israeli young and middle-aged adults, both BMI and ABSI at baseline were positively associated with risk of CHD hospitalization over 20 years of follow-up. ABSI showed a greater increase in risk than BMI per SD, and both associations, especially that of ABSI, persisted after adjustment for sociodemographic and classic CVD risk factors. For both ABSI and BMI, associations with CHD-related hospitalization were stronger for women than for men, but no formal interaction was detected, possibly due to low incidence of CHD-related hospitalizations among young and middle-aged females. Additionally, ABSI, but not BMI, was a significant predictor of all-cause and especially CVD mortality during follow-up. ABSI predicted mortality risk across BMI categories, and in both men and women.

BMI in the obesity range is well-recognized as a risk factor for CVD development. For example, in the Framingham Heart Study cohort, a 10-year CVD risk prediction function that incorporated BMI instead of total and HDL cholesterol, alongside diabetes, smoking, and blood pressure, “performed reasonably well” (16). In a study of Israeli working-age adults, the prevalence of metabolic syndrome components increased linearly with BMI (17). More generally, the Global Burden of Diseases, Injuries, and Risk Factors Study found that higher than optimal BMI is likely to be among the leading contributors to disease burden, particularly in wealthier countries (18). According to the Global Cardiovascular Risk Consortium, underweight, overweight, and obese BMI categories elevate the risk of CVD, particularly for women (19).

Previous studies have compared BMI with measures of central adiposity, including ABSI, for predicting CVD. In a 7-year follow-up of the British Health and Lifestyle Survey, BMI was not a significant predictor of CVD development, whereas the waist-to-height ratio was, but only in men (20). In a Dutch sample, ABSI outperformed BMI and was comparable to cholesterol measurements in predicting 10-year CVD in men, whereas neither BMI nor ABSI predicted CVD well in women (21). In Iran, ABSI outperformed BMI for predicting 10-year CVD in both men and women (22). In a multiethnic cohort study of Inuit and Danes from Canada, Greenland, and Denmark, BMI predicted 10-year CVD somewhat better than ABSI (23). In the Korean Genome and Epidemiology Study, ABSI was a better linear predictor of 10-year CVD than BMI or WC (24). In another analysis of this study, people with BMI-defined obesity who were metabolically healthy at baseline did not have significantly elevated CVD risk, but ABSI could identify people at increased risk within this subgroup (25).

ABSI has also been linked to subclinical cardiovascular markers. It may mediate risk associations with ultrasound-based assessments of vascular structure and function (26, 27), and partially mediate the relationship between smoking and cardio-ankle vascular index (28). ABSI has been associated with incident abdominal aortic calcification (29). In patients with heart failure with preserved ejection fraction, ABSI was found to further stratify prognosis (30). Additionally, various inflammation markers of CVD risk are associated with ABSI (31).

Many studies have assessed mortality as a function of BMI and/or ABSI. For BMI, large studies and meta-analyzes consistently report a U-shaped association with mortality. However, there is some variation across studies regarding the specific BMI value associated with the lowest mortality rate (32, 33). These differences may reflect variation in the level of covariate adjustment (e.g., whether other risk factors such as smoking were accounted for) and differences in population characteristics. For example, among older people and those with serious illness, the optimal BMI tends to be higher, and the adverse effect of elevated BMI is often attenuated—a phenomenon commonly referred to as the “obesity paradox” (34, 35). Mortality risk generally increases monotonically with increasing ABSI, making it a stronger linear mortality predictor than BMI in most studies (36). Its independence from BMI also enables ABSI to complement BMI and achieve better individualized risk assessment by combining information from both indices (7, 37). Conceptually, ABSI complements BMI by its sensitivity to fat distribution and body composition, as elevated ABSI is associated with abdominal and visceral adiposity and with less massive and weaker musculature (38–40). Mortality prediction with ABSI has been confirmed in a large Asian cohort (41), but not confirmed in a much smaller study from China involving middle-aged men followed for 12 years (42). An association between ABSI and mortality has also been reported in studies from Japan (43), Korea (44), and Iran (45). The relationship between ABSI and mortality was confirmed among individuals with normal weight in the Chinese population (46), and similarly in a study from Italy of active non-obese older adults (47). Among those classified with metabolic syndrome in NHANES, considering ABSI additionally improved mortality prediction (41, 48).

The current study complements a previous analysis of the Israeli National Health and Nutrition Survey for Older Adults (≥65 years at baseline), which found that ABSI but not BMI predicted mortality as well as frailty at >10 years follow-up (8). This study extends these previous findings and shows that ABSI predicts mortality risk also among young and middle-aged adults, as well as the risk of CHD-related hospitalization—associations that remain robust after adjustment for sociodemographic and classic CVD risk factors.

### Limitations

Limitations of the current study include a relatively small sample size compared to larger population-based studies. This is particularly important given the younger age of participants, which resulted in fewer deaths and CHD-related hospitalizations during follow-up. In addition, participants were excluded from the current study due to missing data including inability to link to official registry. This can pose a selection bias, and therefore we have compared the excluded and included populations with no substantial differences found (Supplementary Table 1). Additionally, determining previous hospitalizations history based on the registry might lead to misclassification of participants in case the registry is incomplete our prior hospitalizations to the registry. We have added a sensitivity analysis based also on self-reported CHD where the results were consistent with the primary analysis (Supplementary Table 2). Due to the stronger association between CHD-related hospitalizations in female participants, we have assessed for potential interaction between sex and CHD-related hospitalizations and have not found any interaction. This might be due to the lower incidence of CHD-related hospitalizations among female participants, which might limit our statistical power to detect an interaction. Consequently, our ability to identify the exact shape of the association between BMI and mortality, or to quantify differences in risk across subgroups (e.g., by sex), may be limited. In addition, our models were based on a single baseline anthropometric measurement; therefore, any significant changes during follow-up were not accounted for, which may have influenced the results. Another limitation of this study is that some covariates, such as smoking and comorbidities, were self-reported, potentially introducing misclassification bias. Lastly, we used hospitalizations due to CHD as a proxy for CHD incidence, which may have led to outcome misclassification due to incomplete ascertainment.

## Conclusion

In conclusion, in this 20-year prospective study of a national cohort of young and middle-aged adults, ABSI showed a stronger association with mortality and CHD-related hospitalization than BMI and predicted these outcomes independently of BMI and across BMI categories. Further research is needed to provide additional information, including the incremental predictive value of ABSI and its clinical utility in routine clinical assessment and risk stratification, alongside the widespread use of BMI in healthcare settings.

## Data Availability

The data analyzed in this study is subject to the following licenses/restrictions: Survey data are publicly available upon request, whereas outcome data cannot be publicly shared. Requests to access these datasets should be directed to Prof. Yariv Gerber, yarivg@tauex.tau.ac.il.

## Footnotes

1 Cardiovascular diseases 2025. Available from: https://www.who.int/health-topics/cardiovascular-diseases#tab=tab_1.

2 Heart Disease Facts | Heart Disease | CDC 2024. Available from: https://www.cdc.gov/heart-disease/data-research/facts-stats/index.html#libraryItemId=17320102.

3 https://www.gov.il/BlobFolder/reports/mabat-1999-2001-a/en/files_publications_units_ICDC_mabat-25-64-1999-2001-Part1en.pdf.
