# Supplementary material for: Midlife BMI and A Body Shape Index in relation to mortality and coronary heart disease events in a nationwide cohort
Source: Front Nutr. 2026 Sep 1;13:1906316. doi: 10.3389/fnut.2026.1906316 (PMC13574695; doi:10.3389/fnut.2026.1906316)
Supplement: Supplementary file 1 [file Supplementary_Tables_1.DOCX]

**Midlife BMI and A Body Shape Index in Relation to Mortality and Coronary Heart Disease Events in a Nationwide Cohort**

SUPPLEMENTAL INDEX

**Supplemental Tables**

| **Supplemental Table 1.** Baseline characteristics among included and excluded participants | Page 2 |
| --- | --- |
| **Supplemental Table 2.** Hazard ratios for CHD hospitalization per 1 SD increase in BMI and ABSI with self-reported CVD exclusion | Page 2 |

**Supplemental 1.** Baseline characteristics among included and excluded participants

| Factor | Included (n=2225) | Excluded (n=1021) | SMD |
| --- | --- | --- | --- |
| Mean age (SD) | 43.11 (10.98) | 42.19 (11.23) | 0.083 |
| Male sex (%) | 1113 (50) | 432 (42.3) | 0.155 |
| Mean BMI (SD) | 26.93 (4.67) | 26.81 (5.31) | 0.025 |
| Hypertension (%) | 272 (12.6) | 150 (15.7) | 0.089 |
| Hyperlipidemia (%) | 387 (17.4) | 145 (14.5) | 0.081 |
| Diabetes (%) | 141 (6.4) | 64 (6.4) | 0.001 |
| Current smokers (%) | 683 (30.8) | 287 (28.50 | 0.061 |
| Income (%) |  |  | 0.292 |
| High | 31 (1.4) | 7 (0.7) |  |
| Low | 1431 (64.3) | 683 (66.9) |  |
| Mean education years (SD) | 13.04 (3.94) | 12.26 (4.33) | <0.001 |
| Ethnicity (%) |  |  | 0.269 |
| Arab | 481 (21.6) | 265 (26.0) |  |
| Jewish | 1706 (76.7) | 692 (67.8) |  |
| BMI – body mass index; SD – standard deviation; SMD – standardized mean differences | | | |

**Supplemental 2. Hazard ratios for CHD hospitalization per 1 SD increase in BMI and ABSI with self-reported CVD exclusion**

|  | Adjustment | | | | |
| --- | --- | --- | --- | --- | --- |
|  | Sex | + Socioeconomic Factors | + Classic Risk Factors | | + Anthropometrics |
| **CHD Hospitalization – with only prior CHD-related hospitalizations exclusion**  **N=2146** | | | | | |
| **BMI** | 1.28 (1.13-1.46) | 1.21 (1.06-1.38) | | 1.16 (1.01-1.33) | 1.17 (1.02-1.35) |
| **ABSI** | 1.49 (1.29-1.72) | 1.41 (1.23-1.63) | | 1.42 (1.22-1.64) | 1.43 (1.23-1.65) |
| **CHD Hospitalization – with prior CHD-related hospitalizations and self-reported CVD exclusion**  **N=2094** | | | | | |
| **BMI** | 1.33 (1.16-1.52) | 1.26 (1.10-1.44) | | 1.22 (1.06-1.41) | 1.24 (1.07-1.43) |
| **ABSI** | 1.42 (1.22-1.65) | 1.36 (1.17-1.58) | | 1.35 (1.15-1.58) | 1.36 (1.16-1.59) |
| HRs are reported per 1 SD increase (4.666 for BMI; 0.0059 for ABSI).  ABSI – a body shape index; BMI – body mass index; CHD – coronary heart disease. | | | | | |
